# Impact of COVID-19 on hospitalisation for diverse conditions in European countries

**DOI:** 10.1093/eurpub/ckac129.273

**Published:** 2022-10-25

**Authors:** RA Lyons, AE Schmidt, S Aldridge, S Mathis-Edenhofer, F Estupiñán-Romero, M Thissen, M Gissler, L Palmieri, O Majek

**Affiliations:** 1 Population Data Science, Swansea University Medical School, Swansea, UK; 2 Competence Centre for Climate and Health, Austrian National Public Health Institute, Vienna, Austria; 3 Health Care Planning and System Development, Austrian National Public Health Institute, Vienna, Austria; 4 Institute for Health Sciences in Aragon, Zaragoza, Spain; 5 Epidemiology and Health Monitoring, Robert Koch Institute, Berlin, Germany; 6 Finnish Institute for Health and Welfare, Helsinki, Finland; 7 Karolinska Institutet, Stockholm, Sweden; 8 Istituto Superiore di Sanità, Rome, Italy; 9 Institute of Health Information and Statistic, Prague, Czechia; 10 Institute of Biostatistics and Analyses, Masaryk University, Brno, Czechia

## Abstract

**Background:**

The COVID-19 pandemic has had an unprecedented impact on Europe. Health systems came under strain, with non-urgent treatments postponed and resources reserved for treatment of COVID-19 patients. Delayed care seeking has been reported, for fear of infection with SARS-CoV2. Yet, the scale of this impact remains under researched. This study aims to compare indirect effects of the pandemic in a European cross-country study aiming to highlight the potential of Population Health Information Research Infrastructures (www.phiri.eu).

**Methods:**

Focusing on (i) major vascular events (MVE) and (ii) elective surgery for joint replacements (ESJR) as well as (iii) serious trauma this study analyses individual level hospital data in a standardised harmonised data model. We compared pre-pandemic incidence rates (2018-2019) with rates for 2020 and 2021. Analyses are systematically contrasted with SARS CoV2 incidence rates, and policy measures taken based on the OxCGRT index.

**Results:**

A drop in hospital discharge rates was observed during the pandemic in all countries but differing by condition and month. Socio-economic differences also varied by condition. Our evidence suggests that periods of more severe policy measures also correlated with more dramatic drops in regular hospital activities.

**Conclusions:**

Our findings provide new insights on the dramatic level of de-prioritisation of essential services faced by non-COVID-19 patients in Europe. From a public health perspective, hospital escalation plans should be developed early on to avoid negative mid and long-term health and financial consequences of indirect effects. The study demonstrates the tremendous potential in exploiting health information systems in a systematic way across countries and the value of the PHIRI system. Further research should investigate policy trade-offs involved in severe lockdown measures during a pandemic and variations in health service resilience for future pandemic preparedness.

